# Supplementary material for: Overexpression of AtMYB2 Promotes Tolerance to Salt Stress and Accumulations of Tanshinones and Phenolic Acid in Salvia miltiorrhiza
Source: Int J Mol Sci. 2024 Apr 8;25(7):4111. doi: 10.3390/ijms25074111 (PMC11012609; doi:10.3390/ijms25074111)
Supplement: Supplementary file 1 [file ijms-25-04111-s001.zip › ijms-2894996-supplementary.pdf]

Supporting Information

Overexpression of *AtMYB2* promotes tolerance to salt stress and accumulations of tanshinones and phenolic acid in *Salvia miltiorrhiza*

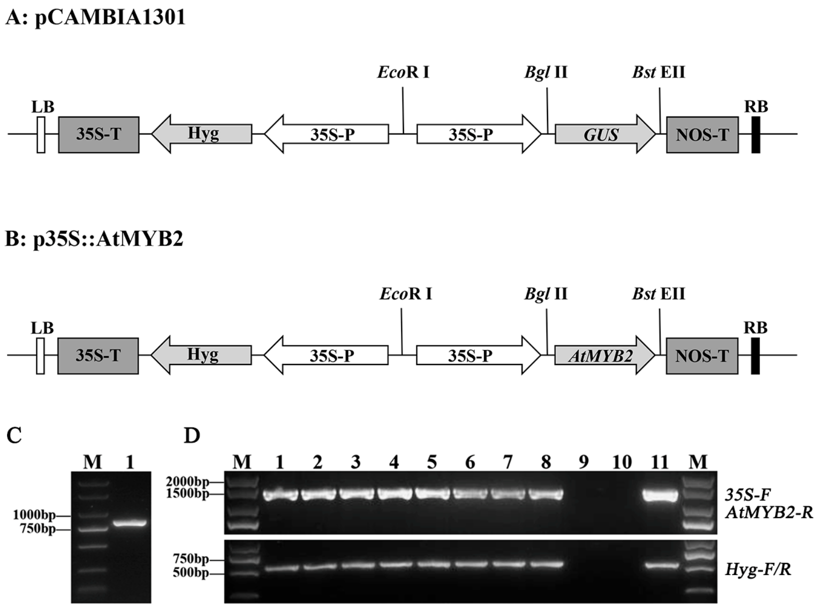

Figure S1. Construction of the *AtMYB2* overexpression vector. A: The schematic diagram of the T-DNA region structure in the pCAMBIA1301 vector; B: The schematic diagram of the T-DNA region structure in the *pCAMBIA1301-AtMYB2* overexpression vector; C: Amplification of the full-length CDS region of the *AtMYB2* gene, M, DL2000 Marker; D: PCR verification of *pCAMBIA1301-AtMYB2* colonies, M, DL2000 Marker, lanes 1-8 represent positive colonies identified by PCR analysis, lane 9, negative control, lane 11, plasmid positive control.

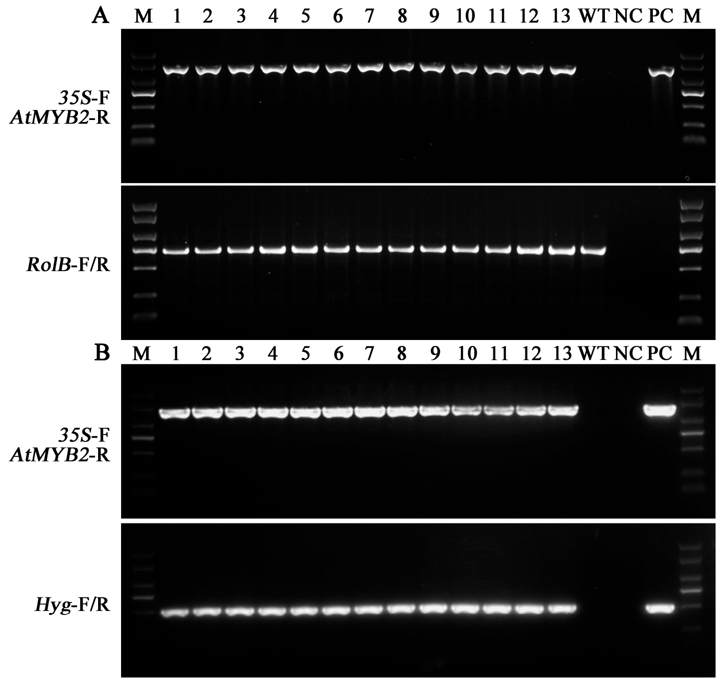

Figure S2. Identification of *AtMYB2* transgenic hairy roots and transgenic plants by PCR. *35S-F*, *AtMYB2-R* primers and *Hyg-F/R* primers were used to identify transgenic positive clones. The *rolB* gene was used to prove that the hairy roots were infected with *Agrobacterium*. WT, wild type; NC, negative control; PC, positive control; M, DL2000 DNA Marker.

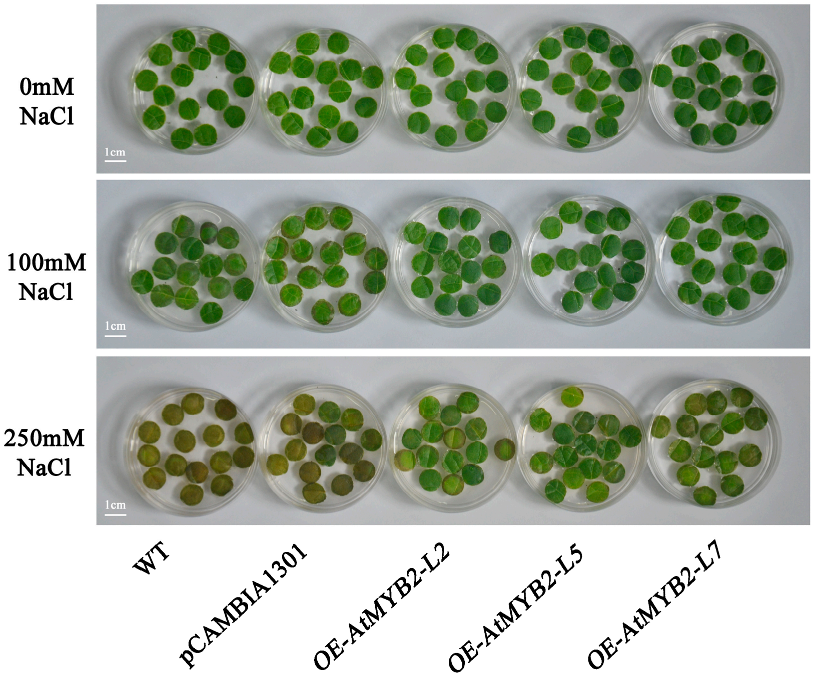

Figure S3. Phenotypic observation of detached leaf discs after treatment with 0 mM, 100 mM and 250 mM NaCl. WT, wild type; pCAMBIA1301, empty vector control pCAMBIA1301 line; *OE-AtMYB2-L2/L5/L7*, *pCAMBIA1301-AtMYB2* transgenic line 2/5/7. Images were taken at 4 DAT (days after treatment).

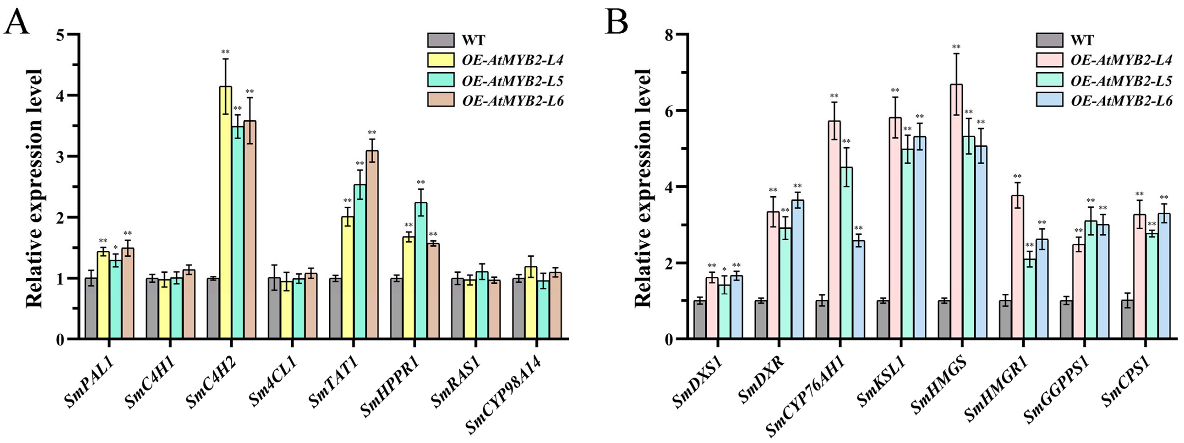

Figure S4. Transcript level of salvianolic acid biosynthesis pathway genes (A) and tanshinone biosynthesis pathway genes (B) in *S. miltiorrhiza* transgenic hairy roots. * and ** represent significant differences at $p < 0.05$ and $p < 0.01$.

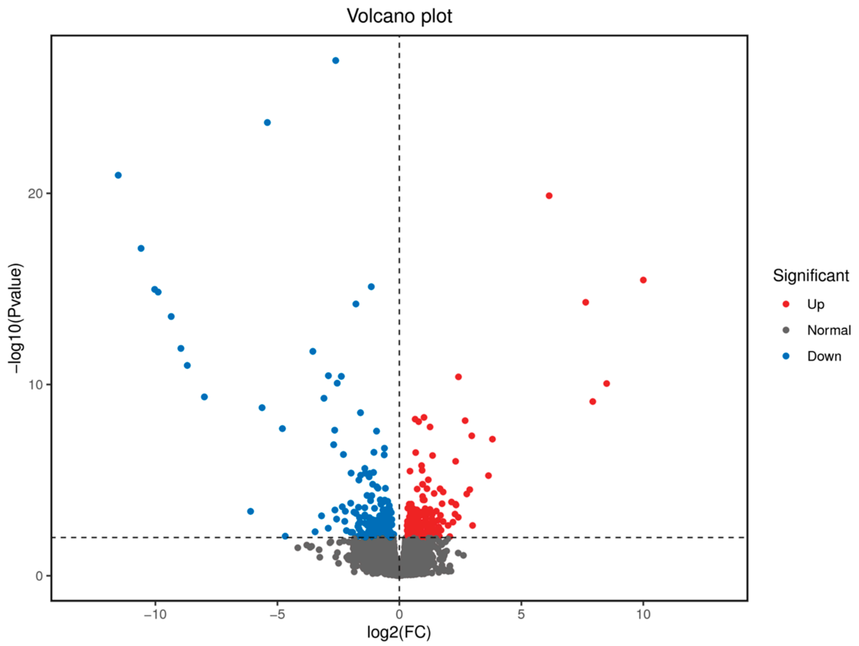

Figure S5. Volcano plot of DEGs of *AtMYB2* transgenic lines versus those of WT plants. Note: in the volcano plot, each dot represents a gene. X-axis: $\log_2$ FC of expression; Y-axis: $-\log_{10}$ (FDR) or $-\log_{10}$ (P-value). Blue dots are down-regulated genes, while red dots are up-regulated ones and black dots are genes without significant differences.

Table S1. Statistics of sequencing data of *AtMYB2* transgenic *S. miltiorrhiza*.

Samples	Clean reads	Clean bases	GC Content	%≥Q30
MYB2-1	20,579,033	6,139,823,990	49.86%	94.00%
MYB2-2	25,998,774	7,769,656,646	50.00%	93.64%
MYB2-3	24,657,193	7,364,060,990	49.19%	94.16%
p1301-1	21,309,595	6,365,646,636	49.66%	93.63%
p1301-2	25,825,132	7,717,456,100	49.38%	94.05%
p1301-3	23,376,367	6,973,266,094	50.92%	94.28%

Table S2. Primers used in this study

Primers	Sequence (5' to 3')
AtMYB2-F	ACTCTTGACCATGGTAGATCTATGGAAGATTACGAGCGAATAA
AtMYB2-R	GGGGAAATTCGAGCTGGTCACCTTAATTATACGAATACGATGTCG
35S-F	AACAGAACTCGCCGTAAAG
Hyg-F	TACACAGCCATCGGTCCAGACG
Hyg-R	TCGGTCAATACACTACATGGCG
rolB-F	GCTCTTGCAGTGCTAGATTT
rolB-R	GAAGGTGCAAGCTACCTCTC
q-AtMYB2-F	CGAATCAACGCCCAATCATTACC
q-AtMYB2-R	ATCATAACCTGACCCGTTCACCA
SmPAL1-QF	ACCCCGTCACCAACCACGTGCAGAG
SmPAL1-QR	CGCCCATTGTGAGAGTTCGTTT
SmPAL2-QF	CAGGATCAAGGGGAGCAGATCGTAT
SmPAL2-QR	CACCATTCCATTCCCTGAGACAG
SmPAL3-QF	CGAGCTCGAGGTGCTCCTC
SmPAL3-QR	CGGCTCTCCATTCCACGATTC
SmC4H1-QF	CCAGGAGTCCAAATAACAGAGC
SmC4H1-QR	GCCACCAAGCGTTCACCAAGAT
SmC4H2-QF	TGGACCATTGCGGAACTCATC
SmC4H2-QR	ATGGAACCACCATTCTACGACG
Sm4CL1-QF	ATTCGCATTCGCATTTCTCGG
Sm4CL1-QR	GCGGCGTAGTGCTTCACCTTT
Sm4CL2-QF	AGCTCTTCATCGTGGACCGA
Sm4CL2-QR	CACGACGAAAGCAACTGGGA
Sm4CL3-QF	GTCGACGGCGACAACCCTAA
Sm4CL3-QR	AGCAGCGAGCCGATCTCAAA
SmTAT1-QF	CAACTGCTGGTCTTCCACAAAC
SmTAT1-QR	GCGAGCCAAAACGGACA
SmTAT2-QF	CGCTGACCCAGCAACCTTTAT
SmTAT2-QR	TGTCATCCTCAATGCCTTCG
SmHPPR1-QF	TGACTCCAGAAACAACCCACATT
SmHPPR1-QR	CCCAGACGACCCTTCACAAG
SmHPPR2-QF	AATGTGGGAGTGATGATGACCAC
SmHPPR2-QR	GCTTGAAGAGTGTGAAGCGC
SmHPPR3-QF	GCGCTACCGCTCCTTGAGAT
SmHPPR3-QR	CACAAATCCGCCGCGAAGTC
SmRAS1-QF	CGAGATCGCCTACTCCAAGTTCAAG
SmRAS1-QR	AGATGGCGTTACCGAAGTATCCCTG
SmRAS-Like-QF	ACCTTGAGTCTAAGCCTGCGTG
SmRAS-Like-QR	CGTCACCCAACTCGTTATCCC
SmCYP98A14-QF	GGTCTGTACCGTCGTCCTCTTCTCC
SmCYP98A14-QR	CCTTTTCCCAAATACCAGCCTTGT
SmDXS1-QF	GGAAGAAGAGACAGGATGCCGAGTT

SmDXS1-QR	AGTCCAGGTAGCCAGCATTGTTCAT
SmDXR-QF	CGACGAGAAAATCGGATACCTGG
SmDXR-QR	CATACAAGAGCAGGACTCAAACCG
SmMCT-QF	AGGTTCTGAAGGATGGCAAGCGAAT
SmMCT-QR	CCATAGCGTCTTCCTGTCCAGTGTT
SmCMK-QF	TACACCAACGCCACTCTTCCTCAA
SmCMK-QR	GCCGTCGCTCTGATAAGATGGATTC
SmMDS-QF	GAGCACGGTTGGAGCAGAGACT
SmMDS-QR	TGAGGAATATTGATGCCGCCGATAATG
SmHDS1-QF	GTTTGGTAGTCTCTTTGCGTGGTG
SmHDS1-QR	AACTGGGAAGTCGAGCGAGTTCTC
SmHDR-QF	TGAAGCTAGAAGACAGTTCCCCTC
SmHDR-QR	GTATTCCAAACCTTAGACACCCATG
SmIDI-QF	GCAACGATCCACAACTAAGGT
SmIDI-QR	ATGCCGAGTTCATCCAACAG
SmGPPS-QF	CACTTTCGCAATCCTATTCAGTC
SmGPPS-QR	GGGAACCTCCGCAACTACCAT
SmGGPPS-QF	AGGTGGAAGCAGCGATCAAAT
SmGGPPS-QR	CCAGCCGTCTTTCCCAACTC
SmCPS1-QF	GATCGCCTCGTCAATACCAT
SmCPS1-QR	TTCGAACCCACAAGTCATGT
SmKSL1-QF	GTGTGACCCTTCTGCTAGCA
SmKSL1-QR	TGCATTGTCTTGGGAAGATG
SmCYP76AH1-QF	TCCATCTCGGCAGCCTCTACAC
SmCYP76AH1-QR	GAGAAGACCTGCCCGTGCCT
SmAACT1-QF	GGAGAGGACGACCATCCACCATTG
SmAACT1-QR	GGCATGAGCGGCATCAGCATATCC
SmHMGR1-QF	TCGTTTTCAATAAGTCGAGTAGA
SmHMGR1-QR	ATTCTGAAGGAAGTCCAAAACAT
SmHMGS-QF	GATGAGCATGACAGCGGTTACTT
SmHMGS-QR	GGATTTGCTCTTGTCGAGTACGGT
SmMK-QF	CTGGGATAGACAATACAGTAAGCAC
SmMK-QR	ACGAGCATCTTCAAAGGCATA
SmPMK-QF	GCTTGCCATTGACCTCGGAATCT
SmPMK-QR	ACCACTGAACTCTGAAGGAAGACTGA
SmMDC-QF	AAGCACTGGGATGACCTCGTTAT
SmMDC-QR	ATGGAACTGATTACTATCGGCACAAG
SmFPPS-QF	GTGGACTGATGGTTCTCGTCAATGG
SmFPPS-QR	CACACCAGCCAAGAGCACTAGC
SmActin-QF	AGCACCGAGCAGCATGAAGATT
SmActin-QR	AGCAAAGCAGCGAACGAAGAGT
